# The caudal regeneration blastema is an accumulation of rapidly proliferating stem cells in the flatworm *Macrostomum lignano*

**DOI:** 10.1186/1471-213X-9-41

**Published:** 2009-07-15

**Authors:** Bernhard Egger, Robert Gschwentner, Michael W Hess, Katharina T Nimeth, Zbigniew Adamski, Maxime Willems, Reinhard Rieger, Willi Salvenmoser

**Affiliations:** 1 Institute of Zoology, Center for Molecular Biosciences, University of Innsbruck, Innsbruck, Austria; 2 Division of Histology and Embryology, Innsbruck Medical University, Innsbruck, Austria; 3 Department of Animal Physiology, Institute of Experimental Biology, Faculty of Biology, Adam Mickiewicz University, Poznañ, Poland; 4 Nematology Section, Department of Biology, Ghent University, Ghent, Belgium

## Abstract

**Background:**

*Macrostomum lignano *is a small free-living flatworm capable of regenerating all body parts posterior of the pharynx and anterior to the brain. We quantified the cellular composition of the caudal-most body region, the tail plate, and investigated regeneration of the tail plate *in vivo *and in semithin sections labeled with bromodeoxyuridine, a marker for stem cells (neoblasts) in S-phase.

**Results:**

The tail plate accomodates the male genital apparatus and consists of about 3,100 cells, about half of which are epidermal cells. A distinct regeneration blastema, characterized by a local accumulation of rapidly proliferating neoblasts and consisting of about 420 cells (excluding epidermal cells), was formed 24 hours after amputation. Differentiated cells in the blastema were observed two days after amputation (with about 920 blastema cells), while the male genital apparatus required four to five days for full differentiation. At all time points, mitoses were found within the blastema. At the place of organ differentiation, neoblasts did not replicate or divide. After three days, the blastema was made of about 1420 cells and gradually transformed into organ primordia, while the proliferation rate decreased. The cell number of the tail plate, including about 960 epidermal cells, was restored to 75% at this time point.

**Conclusion:**

Regeneration after artificial amputation of the tail plate of adult specimens of *Macrostomum lignano *involves wound healing and the formation of a regeneration blastema. Neoblasts undergo extensive proliferation within the blastema. Proliferation patterns of S-phase neoblasts indicate that neoblasts are either determined to follow a specific cell fate not before, but after going through S-phase, or that they can be redetermined after S-phase. In pulse-chase experiments, dispersed distribution of label suggests that S-phase labeled progenitor cells of the male genital apparatus undergo further proliferation before differentiation, in contrast to progenitor cells of epidermal cells. Mitotic activity and proliferation within the blastema is a feature of *M. lignano *shared with many other regenerating animals.

## Background

A single type of cells in flatworms is responsible for regeneration, growth and tissue maintenance, the so-called neoblasts. These likely totipotent stem cells have a high nucleus to cytoplasm ratio, are the only dividing cells in juveniles and adults and have been shown to differentiate into diverse cell types [1-3].

The formation of a regeneration blastema is a response to amputation involving mainly cell proliferation, and describes the accumulation of undifferentiated or dedifferentiating cells at the wound site, often visible as an unpigmented bulge in an otherwise pigmented animal. Blastemas have not only been described in various flatworms [4], but also in annelids [5], molluscs [6], nemerteans [7], echinoderms [8], crustaceans [9], teleost fish [10], urodele amphibians [11], larval anuran amphibians [12], lizards [13] and some mammalians [14].

Even though the formation of a regeneration blastema after amputation or fission is a common occurrence in many bilaterians, flatworms show some peculiarities. One notable difference compared to vertebrates is that in flatworms seemingly no dedifferentiated cells contribute to the formation of the blastema, although redetermination of already determined stem cells may take place [15,16]. Another specialty of the blastema in triclad flatworms (usually referred to as "planarians") is a high mitotic activity at the border between blastema and differentiated tissue, the so-called postblastema [17].

Within flatworms, blastema formation is described in acoels [3,18], polyclads [19], triclads [20] and some – but not all – macrostomorphans [21,22]. In catenulids, stem cells accumulate at the wound site, but these cells are split up into two groups, and are not forming a single blastema [23]. Regeneration processes in rhabdocoels involve a small buildup of "cells of embryonic character" at the wound site [24] and can be interpreted as blastema formation.

Two species of the Macrostomorpha have been examined more closely: Head regenerates of *Microstomum lineare*, a species reproducing asexually by paratomy, show a similar distribution of S-phase cells as controls, and do not form a blastema [21]. Contrary, regeneration in *Macrostomum lignano *involves the formation of a blastema, characterized morphologically by a bulge of unpigmented cells at the wound site [22,25-27].

A large number of studies about regeneration and blastema properties in flatworms was undertaken with triclads, which have been chosen for their remarkable regeneration capacity, availability and their manageable size. However, their comparable large size complicates the quantification of a regeneration blastema, and labeling of S-phase neoblasts with the thymidine analogue bromodeoxyuridine (BrdU) was made possible only by supplying food containing BrdU or with micro-injections [2], and not by simple soaking in a BrdU solution as in *M. lignano *[1]. Only recently, a soaking method was reported to be successful in the triclad *Dugesia japonica*, although BrdU incubation was prolonged for six hours instead of a 30 minute pulse [28]. For these reasons, we have chosen the small flatworm *Macrostomum lignano *with a limited, but well-known regeneration capacity [26,27] and a thoroughly documented and easily stainable stem cell system [1,22,26,27,29-31]. Thanks to its small size and its transparency, cells and organ systems can also be studied *in vivo *squeeze preparations.

In this study we quantified and categorized the tail plate, a body region in the caudal-most part of the flatworm *M. lignano *before and for the first three days after amputation and analyzed cell proliferation, the differentation of cell types and the build-up of organ primordia within the regeneration blastema.

## Methods

### Animals

**Figure 1:**
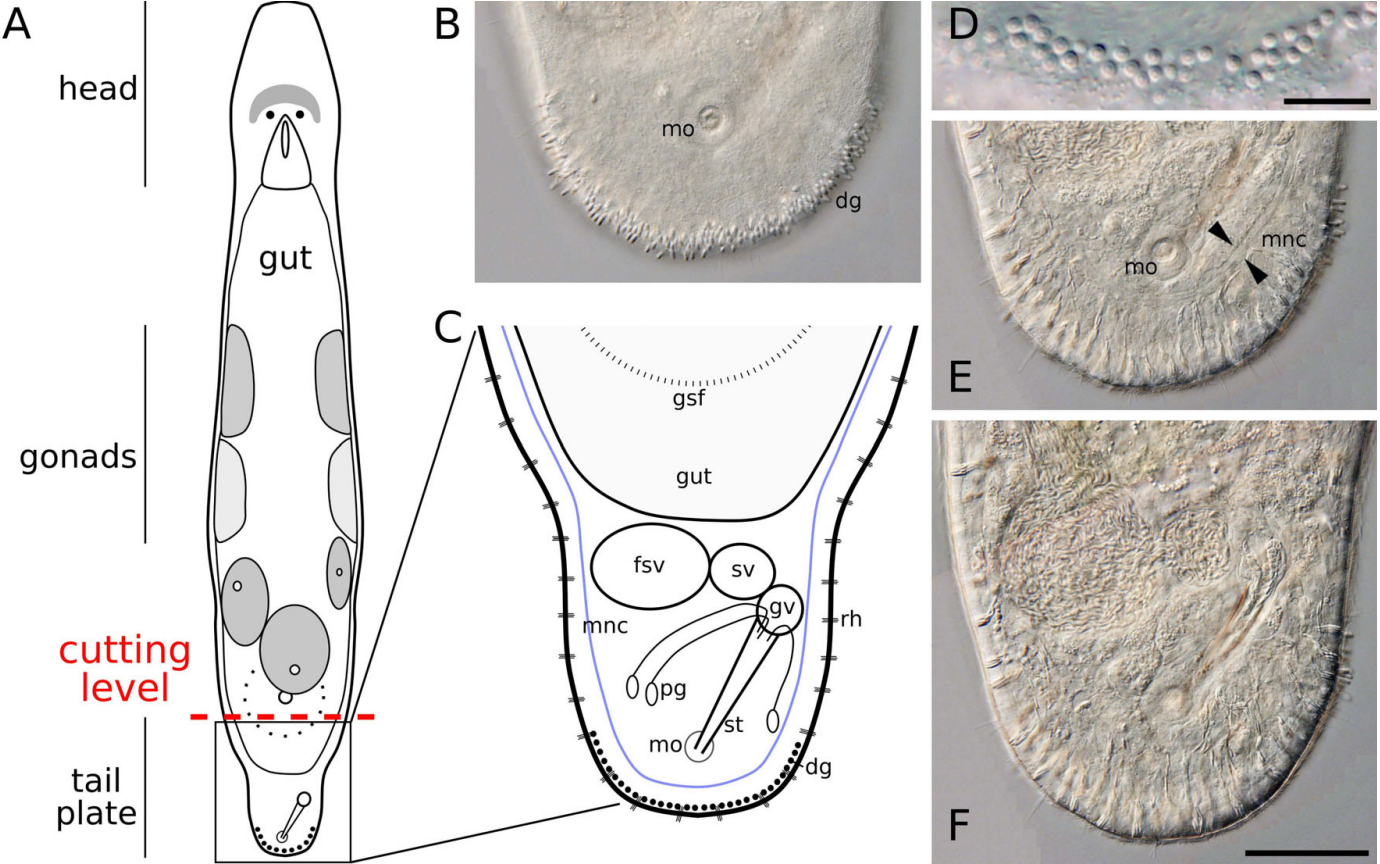
**Overview of the animal *M. lignano***. (A) Schematic drawing of *M. lignano *with indication of the amputation level (red dashed line). Dorsal view. The amputated tail plate is magnified in (C). (B, E, F) Focal interference contrast series through the tail plate of a living specimen. (B) Ventral focus plane showing the male genital opening *mo *and about 140 duo-gland adhesive systems *dg*. (C) Schematic drawing of the tail plate. Attached to the male copulatory organ (stylet *st*) are the vesicula granulorum *gv*, connected with the seminal vesicle *sv *and the false seminal vesicle *fsv*. Prostate glands *pg *are reaching into the *gv *and further down the *st*. Rhabdites *rh *are located between the epidermal cells. *Dg *are emerging at the posterior tip of the tail plate. The posterior end of the sac-like gut and cement/shell glands surrounding the female genital opening *gsf *are at the anterior border of the amputated piece. Main longitudinal nerve cords *mnc *in blue. (D) Higher magnification of *dg*. Scale bar is 10 μm. (E) *Mnc *in focus (band between arrow heads). (F) Mid-level focus plane with the male genital apparatus. Scale bar is 50 μm. (B, E, F) same scale.

The marine flatworm *Macrostomum lignano *reaches 1 to 1.5 mm in length and lays eggs in laboratory cultures all year round. Sexual maturity is reached about two weeks after hatching [32]. This flatworm species can be kept in cultures using the diatom *Nitzschia curvilineata *as food. Animals were raised from eggs and were between three and six weeks old when used for regeneration experiments. Using standard animals of a known and narrow age range avoids age-related problems such as a possibly changing physiology and an increased size. For regeneration studies, the tail plate was amputated with a Gillette razor blade (Fig. 1A). Detailed culture conditions of untreated animals and regenerates are provided in [33].

### *In vivo *observations

Live squeeze preparations were made according to [34]. Animals anesthetized in a 2:1 mixture of 7.14% MgCl_2_.6H_2_O and artificial seawater (ASW) were observed with interference contrast (Nomarski) microscopy. Measurements of blastema extension were done using pictures of slightly squeezed live animals. For staining of epidermal nuclei, living specimens were treated for 4 minutes with Hoechst 33342 (Sigma-Aldrich) at a concentration of 1:1000 diluted in ASW and then washed thoroughly in ASW. Anesthetized and squeezed animals were observed with fluorescence microscopy. In cell macerations and in fixed animals, Hoechst 33342 unspecifically stains all nuclei, but only epidermal nuclei in intact *M. lignano*. The chemical is not acutely toxic for the animals, which have been found to survive the treatment for at least seven days (personal observation).

### Staining of fixed animals (whole mounts)

Animals were anesthetized in 7.14% MgCl_2_.6H_2_O for 5–10 min, fixed in 4% formaldehyde (FA) for 1 hour and washed with phosphate buffer saline with 0.1% Triton (PBS-T) for 10 minutes. Muscle F-actin was revealed using Alexa Fluor 488 phalloidin (Molecular Probes) diluted 1:200 in PBS-T, staining for 1 hour in the dark. For staining of the GYIRFamidergic nervous system, following fixation in FA the animals were washed in PBS-T for 30 min, in BSA-T (add 1% Albumin fraction V, Merck, to PBS-T) for 30 min, then incubated over night in a 1:500 solution of neat GYIRFamide antiserum in PBS at 4°C. The next day, animals were washed in PBS-T several times, in BSA-T for 15 min and then incubated in FITC-conjugated goat anti-guinea pig (DAKO) 1:200 in BSA-T for 1 hour. For labeling of S-phase nuclei, live animals were soaked in a 5 mM solution of 5-bromo-2'-deoxyuridine (BrdU, Sigma-Aldrich) in ASW for 30 min. In BrdU pulse experiments, soaking was performed just before anesthetization and fixation. In BrdU pulse-chase experiments, soaking was performed before amputation to allow for migration and differentiation of labeled cells during regeneration time. After fixation with 4% FA and the washing steps in PBS-T, animals were incubated in 0.15 mg/ml Protease XIV (Sigma-Aldrich) for about 20 min (until the epidermis appeared as a jagged instead of a smooth line) at 37°C and then treated with 0.1 M HCl (on ice) for 10 min. Specimens were then incubated in 2 M HCl at 37°C for 1 hour, rinsed in PBS, incubated in BSA-T for 30 min and then in primary mouse anti-BrdU antibody (1:600 in BSA-T, DAKO) over night at 4°C. Subsequently, animals were rinsed in PBS and then incubated in secondary goat anti-mouse antibody (FITC-conjugated, 1:150 in BSA-T, DAKO) for 1 hour for fluorescence labeling. For fluorescent double-labeling of S-phase cells with mitoses, a rabbit anti-phos-H3 antibody (Upstate Biotechnology) was added to the primary antibody solution in a concentration of 1:150, and visualized with a TRITC-conjugated swine-anti-rabbit antibody (Dako) 1:150 in the secondary antibody solution. For all stainings above, animals were washed in PBS after the last antibody incubation and mounted in Vectashield (Vector Labs). For permanent non-fluorescent labeling, instead of a FITC-conjugated secondary antibody, a biotinylated goat anti-mouse antibody was used to allow for the binding of a streptavidin and biotinylated horseradish peroxidase complex (StreptABComplex/HRP Duet, Mouse/Rabbit, DAKO), that was visualized with a chromogenic peroxidase substrate solution (DAB+ Chromogen, DakoCytomation). Concentrations were used according to manufacturer's instructions.

### Tissue macerations

The tail plates of 20 adult specimens relaxed in MgCl_2 _were amputated and 10 tail plates each were transferred to 50 μl of calcium- and magnesium-free medium with 1% trypsin and incubated for 1 hour at 37°C. 50 μl of glycerol:glacial acetic acid:distilled water (1:1:6.5) and Hoechst 33342 for a final concentration of 1:1000 were added and the solution was gently pipetted up and down for about 30 min to obtain single cells. Cell countings were made with a Bürker's hemocytometer on a Leica DM 5000 fluorescent microscope.

### Semithin sections

Animals treated with BrdU, visualized by horseradish peroxidase/DAB+ Chromogen (see above), were dehydrated in a graded series of methanol, transferred in a 1:1 mixture of methanol and Spurr's resin [35] for at least 1 hour and embedded in Spurr's resin.

Untreated animals were fixed in 2.5% glutaraldehyde in 0.1 M cacodylate buffer with 9% sucrose for one hour and postfixed with 1% OsO4 in 0.1 M cacodylate buffer for one hour, dehydrated in a graded acetone series and embedded in Epon-Araldit [36] or Spurr's low viscosity resin.

Embedded specimens were cut for sagittal, horizontal and cross sections at 1 μm thickness with a Reichert Jung 2040 microtome and stained with methylene blue or Heidenhain's iron hematoxyline [37].

### Microscopy and photographs

For live squeeze preparations, stained whole mounts and sections, a Reichert Jung Polyvar and a Leica DM 5000 light microscope were used. Photographs were taken with a Pixera Penguin 600CL and a The Imaging Source DFK 41F02 camera. Picture editing and drawing schemes was done with the free programs Inkscape [38] up to version 0.45 and GIMP [39] up to version 2.2.13. Some stained whole mounts were observed with confocal Zeiss LSM 510 and 710 microscopes.

### Cryo-based transmission electron microscopy

High-pressure freezing and freeze-substitution were used to complement and evaluate data from conventional chemical fixation [40]. Briefly, live animals in their culture medium were pipetted into cup-shaped aluminium specimen holders of a HPM010 high-pressure freezing apparatus (BAL-TEC, Balzers, Liechtenstein) and rapidly frozen at a pressure of approximately 2100 bar. Freeze-substitution was carried out with anhydrous acetone supplemented with 1% OsO_4 _and 0.2% uranyl acetate (at -90°C, 18 h), followed by Epon embedding. Semithin and ultrathin sections were stained according to standard procedures and viewed by brightfield light microscopy or transmission electron microscopy (with a CM120, FEI-Philips), respectively.

### Blastema cell counts

The total blastema cell number and the percentage of BrdU-labeled and unlabeled cells was counted from semithin serial sections. In all sections containing a part of the blastema, all cells or nuclei were counted. In sections of animals labeled with BrdU (pulse or pulse-chase, see staining procedures), both labeled and unlabeled cells (nuclei) were counted.

Subsequently, the average blastema cell dimension was determined, i.e., through how many sections a single cell could be followed. This was done for 15–20 cells (separately for labeled and unlabeled cells) for each blastema. The total number of blastema cells was calculated by dividing the number of counted cells by the average blastema cell dimension, both for labeled and unlabeled cells. Epidermal cells covering the blastema were not included in blastema cell counts.

## Results

### Cell numbers in the tail plate of intact animals

**Figure 2:**
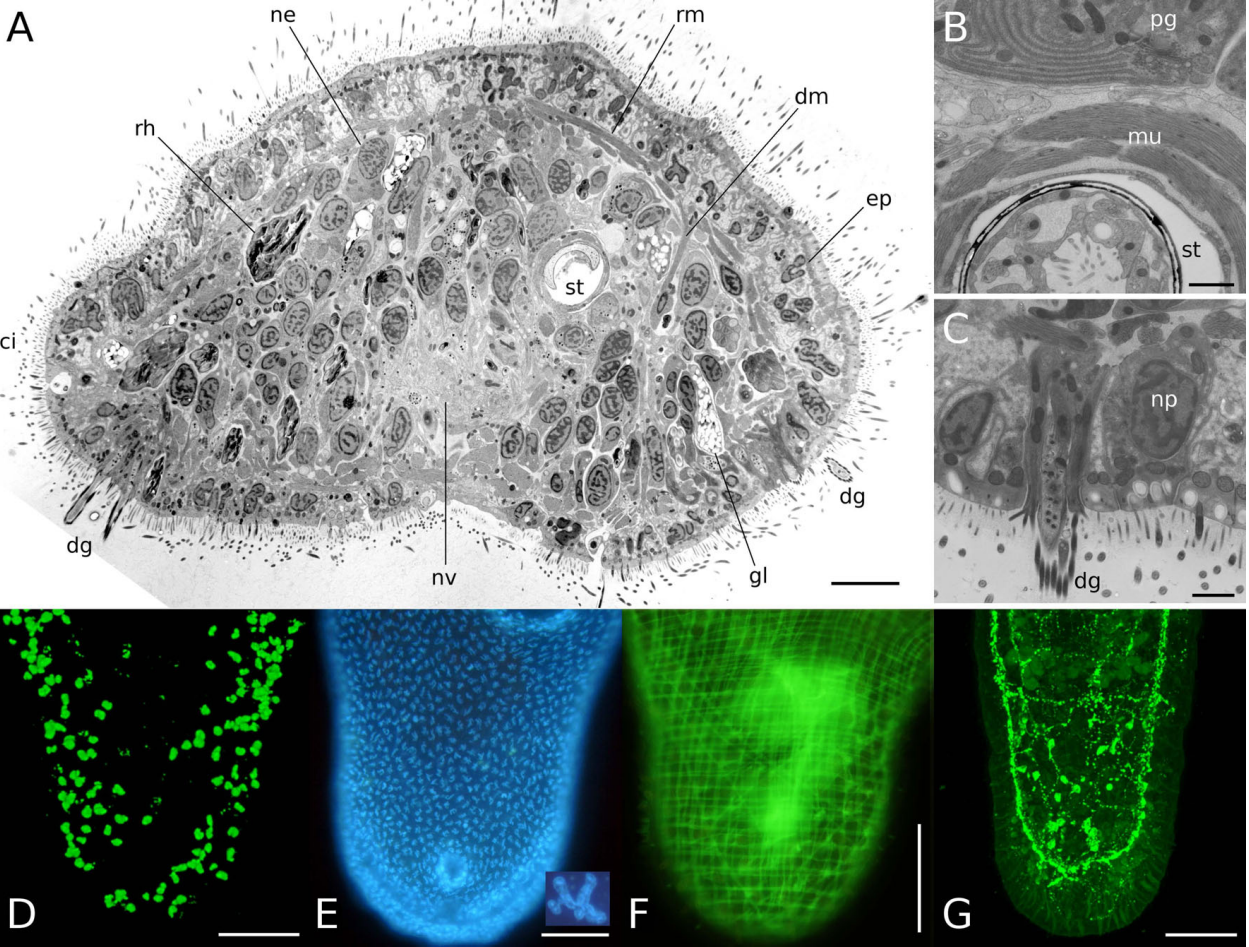
**The tail plate of *M. lignano***. (A-C) Electron micrograph of an adult specimen fixed with high pressure freezing. (A) Cross section through the tail plate at the tip of the stylet with representative cell types. Dorsal side is up. (B, C) Details of a more anterior tail plate section than (A). (B) The stylet is surrounded by musculature. The inner epithelial lining is interspersed by muscle cells as well. (C) The tip of a duo-gland adhesive system besides a new epidermis cell in differentiation. *ci *cilia *dg *duo-gland adhesive systems, *dm *dorsoventral muscles, *ep *multiciliated epidermal cell, *gl *cyanophil gland cell, *mu *muscle fibers, *ne *neoblast, *np *new epidermal cell, *nv *nerve, *pg *prostatic gland, *rh *rhabdite gland, *rm *ring muscles, *st *stylet. (D-G) Fluorescent whole mount stainings of the tail plate. (D) Neoblasts in S-phase labeled with BrdU, (E) epidermal nuclei (inset shows a single lobulated epidermal nucleus) labeled with Hoechst 33342, (F) muscle F-actin stained with phalloidin, (G) GYIRF-amidergic nervous system. Scale bar in (A) is 5 μm, in (B, C) 1 μm, in (D-G) 100 μm.

The tail plate was defined as the body region between the posterior end of the shell/cement gland ring surrounding the female genital opening and the caudal tip of the animal (Fig. 1A, C). This region can be easily identified under a dissection binocular before and after amputation, ensuring a comparable cutting level in all amputated individuals and a relatively small size of amputated tissue. The same amputation level was used in a previous study [22]. To determine the cell number of the tail plate, different staining and visualisation techniques specific for the various cell types were applied. Cell numbers of cell types that were not specifically stained are based on estimations from whole mounts and sections. A representative ultrathin cross section of the tail plate at the tip of the stylet is shown in Fig. 2A, and more detailed views in Fig. 2B, C. An overview of all cell counts is given in Tab. 1.

**Table 1:** Cell types and numbers in the tail plate

***Cell type***	***Number***	***%***
Epidermal cells	1,575	51
Duo-gland adhesive systems	390	13
Other gland cells	197	6
Neoblasts	355	11
Muscle cells	183	6
Nerve cells (GYIRFamid)	22	1
Gut cells	10	0
Male genital apparatus	78	3
Sperm	100	3
Other cell types	200	6
Total	3,110	100

Number of cells per cell type in the tail plate of adult *M. lignano*, based on averages of countings and estimations (gut cells, "other cell types")

Nuclei of all epidermal cells were stained in living specimens with Hoechst 33342, which does not penetrate the epidermal layer in live animals and therefore specifically labels epidermal cells (Fig. 2E). The dorsal or the ventral cell number was counted up to the female genital opening and then multiplied by 2, as both sides were found to give similar cell numbers. The average number of epidermal cells posterior of the female genital opening was 1,575 ± 136 (n = 9). In TEM cross sections, 40–50% of all cells can be identified as epidermal cells (Fig. 2A), corroborating the fraction of epidermal cells found in Hoechst stainings.

The duo-gland adhesive systems are located at the posterior ventral tip of the tail plate, identifiable as rings protruding from the surface (Figs. 1B, D, 2A). They consist each of two gland cells and an anchor cell [41]. The average number of duo-gland adhesive systems in standard animals was found to be 130 ± 17 (n = 13) for a total of 390 cells by multiplication with 3. Rhabdite glands were counted in live squeeze preparations for a total of 56 ± 6 (n = 9) on the ventral or dorsal side of the tail plate. Comparable numbers were obtained for the dorsal and the ventral side. Accounting for both sides gives 112 rhabdites on average per tail plate. The number of prostate glands in the tail plate of adult *M. lignano *is reported to be up to 35, found by labeling with a monoclonal antibody [42]. Cyanophilic ("slime") glands (for their appearance see Fig. 2A) account for about 50 cells, inferred from ultrathin sections (Rieger, unpublished observations), adding up to about 197 gland cells other than duo-gland adhesive systems in the tail plate.

BrdU-labeling revealed the average number of S-phase cells in the tail plate to be 96 ± 29 (n = 15, Fig. 2D). Bode et al. [30] report that S-phase cells account for about 27% of the total neoblast population, rendering the average number of neoblasts in the tail plate to be 355 cells.

Muscle cells in the tail plate area were revealed by phalloidin staining. The muscle rings counted from the ventral (n = 7) or the dorsal (n = 5) side gave an average of 48 ± 7 (n = 12) muscle rings – often closely associated in pairs – per tail plate (Fig. 2F). As is known from phalloidin-rhodamine preparations and electron micrographs of *M. hystricinum marinum *[43], one muscle ring is composed of about 3 ring muscle cells, so that the calculated average number of ring muscle cells per tail plate is 144. The number of dorsoventral muscle cells in the tail plate of adult *M. lignano *is reported to be 40 [44]. From the available data, the approximate number of muscle cells in the tail plate of *M. lignano *can be estimated as 183. Longitudinal muscles were not considered in the present work, because previous studies have shown that during regeneration most longitudinal fibers are elongated without build-up of new cell bodies [25]. Diagonal fibers in *Macrostomum *are known to branch off of longitudinal fibers [43] and were not counted as well.

The number of nerve cells contributing to the tail plate was calculated from GYIRFamidergic (Fig. 2G) nervous system stainings in adult animals. Analyzing confocal stacks, the average number of GYIRFamidergic nervous cells was found to be 22 ± 1 (n = 5).

Some gut cells are cut off when amputating the animals posterior of the female genital opening. Being one of the largest cell types in *M. lignano*, the amputated number of these cells was estimated to be approximately 10 cells, inferred from semithin and TEM sections (data not shown).

The male genital apparatus is made up of the copulatory organ (stylet) and the vesicula granulorum, the seminal vesicle and the false seminal vesicle, each attached to the previous structure (Fig. 1C, F). About 35 prostate glands (see gland cells) reach into the stylet, which consists of about 8 cells and is lined with about 10 epithelial cells. The granular and seminal vesicles are lined with about 10 epithelial and 10 muscle cells, and 5 epithelial and 15 mucle cells, respectively. About 10 ring muscle cells (Fig. 2B) and 10 longitudinal muscle cells contribute to the musculature of the stylet. These estimations were obtained from TEM sections of another *Macrostomum *species, *M. hystricinum marinum *(Rieger, unpublished observations). All in all, about 78 cells make up the male genital apparatus (excluding prostate glands and sperm found in the seminal vesicles).

The sperm number in the tail plate may vary, depending on copulation frequency. Isolated worms tend to accumulate sperm in the seminal vesicles. An estimate of the average sperm number in the seminal vesicle and the false seminal vesicle is about 100 from live squeeze preparations, as well as from a confocal stack of Hoechst 33342-labeled sperm nuclei in the seminal vesicles.

Parenchyma cells, different kinds of nerve cells and other, unidentified cell types are estimated to comprise up to 200 cells in the tail plate.

The total number of cells in the tail plate amounts to about 3,110 cells (Tab. 1), about half of which are epidermal cells. In tissue macerations of 20 tail plates, 3,176 ± 40 cells with Hoechst-33342-labeled nuclei were counted in 21 grids of 1 mm^2 ^using a hemocytometer, corroborating the number of about 3,100 cells given above. While not all cell numbers are equally accurate, they may indicate which cell types and numbers are to be regenerated after amputation.

### The tail plate blastema

**Figure 3:**
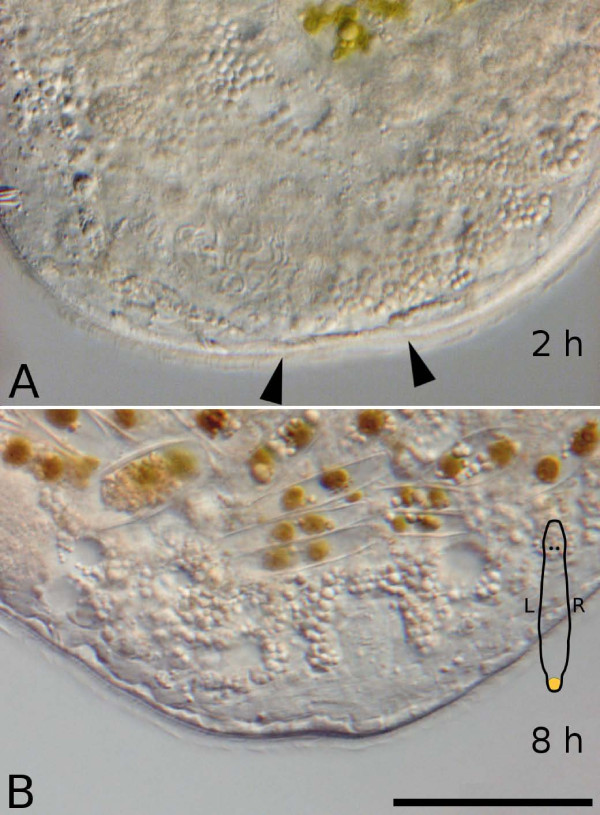
**Early stages of wound closure and healing after amputation**. Interference contrast pictures of living specimens.

To study the formation and the development of the tail plate blastema, specimens were transversally amputated posterior of the female genital opening (Fig. 1A). Immediately after amputation, animals contracted their circular musculature near the amputation site to minimize the open wound surface (see also [25]). The longitudinal musculature was also contracted, even more so on the ventral than on the dorsal side, so that the wound surface and the early blastema were bent to the ventral side. Epidermal cells in the wound area flattened to cover the wound surface (Fig. 3). The blastema remained in a ventral position for approximately 24 hours after amputation. Only between 24 and 28 hours after amputation the ventral longitudinal muscles relaxed to allow the blastema taking a more caudal position (4A, B, 5A, B). In cross sections and live squeze preparations, the blastema was clearly visible after 24 hours of regeneration and later, distinctly set apart from the bordering gut and the female gonads.

In the first four to six hours after cutting, the wound closure was still fragile and would sometimes break open in slightly squeezed animals anesthetized with MgCl_2_. After about 8 hours enough epidermal cells surrounding the wound flattened to cover the whole wound area (Fig. 3B).

The blastema was examined *in vivo *and in serial sagittal sections at three different time points: 24, 48 and 72 hours after amputation.

#### 24 hours after amputation

**Figure 4:**
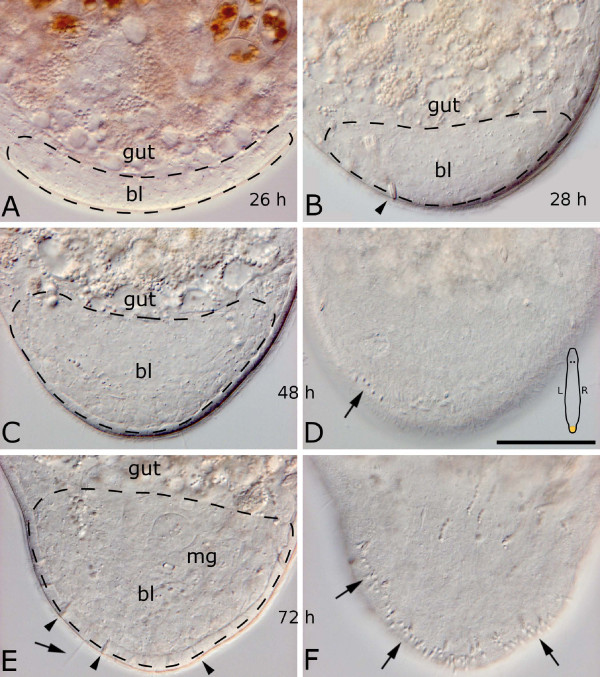
**Interference contrast images of a tail plate blastema of living specimens**. 26 (A), 28 (B), 48 (C, D) and 72 (E, F) hours after cutting. The scheme in (D) is indicating the orientation of the animals in all subpanels. (C, D) and (E, F) are different focal planes of the same specimens, respectively. The blastema *bl *makes up the posterior part of the body and is delimited anteriorly by the gut. 24–28 hours after cutting, the blastema shifts from its ventral position (A) to the posterior end of the body (B). (C) 48 hours after cutting the blastema has grown substantially, and several duo-gland adhesive systems have been rebuilt (arrow in D), which significantly increase in number after 72 hours (F, arrows). At this time, the male genital apparatus *mg *is being rebuilt with the first tip of the stylet and a small vesicula granulorum to be seen. An arrow points at a long sensory cilium (E). Arrowheads denote rhabdites. Dotted lines indicate blastema extension. Scale bar is 50 μm for all sub-panels.

**Figure 5:**
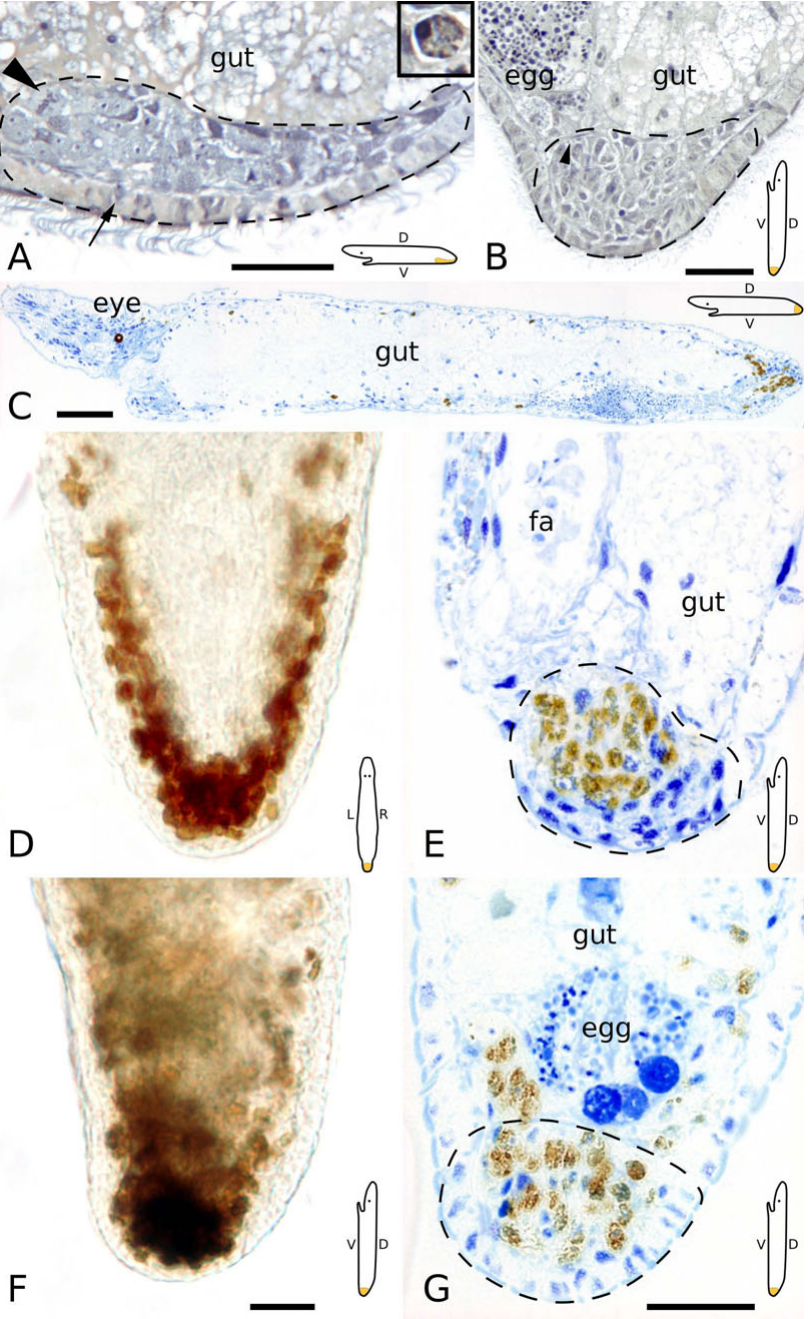
**Semithin sections and whole mounts of 24 hour tail plate blastemas**. Schemes are indicating the orientation of the animal in the respective subpanels. (A) Blastema is still located ventrally. Note the mitosis at the anterior end of the blastema (arrowhead). The newly built epidermal layer is characterized through smaller, often less ciliated cells (area above scale bar). A cell migrating to the epidermal layer is shown by the arrow. (B) Blastema is already located caudally. Arrowhead denotes a dorsoventral muscle fiber. (A, B) stained after Heidenhain, (C, E, G) with methylene blue. (C, D, E) BrdU pulse (brown) was applied just before fixation. All three subpanels from the same animal. (C) Complete section, anterior is left. Note the accumulation of labeled neoblasts in the caudal blastema (right). (D) Whole mount and (E) section of the blastema, with accumulation of neoblasts. *fa *female antrum. (F, G) BrdU pulse chase experiment. Both subpanels are from the same animal. Note the accumulation of labeled cells in the blastema, but the absence of labeled cells in the epidermis. Dotted lines indicate blastema extension. All scale bars are 20 μm, except 50 μm in (C). (D, F) and (E, G) in the same scale.

On average, the 24 hour blastema was made of about 420 cells (423 ± 67, n = 6), counted from serial sections, not including epidermal cells. Depending on whether the blastema was still located ventrally or already in a caudal position (Fig. 4A, B), its shape in sagittal sections was either approximately that of an elongated drop (Fig. 5A), or of a circle (Fig. 5B). Located ventrally, the maximal elongation in the anterior-posterior axis reached about 90 μm in sections (n = 2). In its caudal position, the blastema extended 37 ± 8 μm in the anterio-posterior, 51 ± 14 μm in the dorso-ventral, and 34 ± 7 μm in the left-right axis in sections (n = 4).

An average of 5 ± 3 mitoses (n = 17) was identified in the blastema region in serial sections (Fig. 5A) and fluorescent confocal stacks (see Additional file 1A). Most of the mitoses were located at the anterior-most border (27%) and in the middle of the blastema (56%), but some mitoses were also found in the caudal-most part of the blastema (17%), even bordering epidermal cells at the posterior tip. About one tenth (9%) of all mitoses were situated in the dorsal third of the blastema. In BrdU pulse serial sections an average of 178 ± 36 (n = 2) S-phase cells was counted in the blastema, which made up 37% of all cells in the blastema (Fig. 5C–E). A majority of S-phase cells was found in the ventral and middle area of the blastema (Fig. 5E), only about one fifth (22%) of labeled cells were located in the dorsal blastema region. In BrdU pulse-chase experiments more than 200 cells were labeled with BrdU (210 ± 28, n = 2), constituting 58% of all blastema cells. Labeled cells were distributed evenly in the blastema (Fig. 5F, G).

New epidermal cells, identifiable by darker (ribosome-rich) cytoplasm, a round instead of a lobulated nucleus (Fig. 2C) and shorter cilia (see also Additional file 2), migrated to the wound area to replace lost epidermis (Fig. 5A), although labeled epidermal nuclei were not yet found in 24 hours BrdU pulse-chase experiments (Fig. 5F, G). Longitudinal and also circular muscle fibers could be detected in the blastema region. The main lateral nerve cords were already reconnected in a caudal loop (data not shown).

At this stage, several gland cells were already present in the blastema. Although rhabdite glands were predominantly found at the ventral and dorsal border of the blastema, sporadically they were also located at the posterior blastema border (Fig. 4B). Duo-gland adhesive systems were neither detected in sections nor in live squeeze preparations (Fig. 4A, B). Some dorsoventral muscle fibers were already restored (Fig. 5B). Necrotic cells by injury or apoptosis could be identified in serial sections, predominantly located at the anterior border of the blastema (Fig 5A inset). Cells within the blastema generally had a large nucleus and a prominent nucleolus and varied mainly in the staining intensity, with color ranging from light to dark blue in sections stained after Heidenhain (Fig. 5A, B).

#### 48 hours after amputation

**Figure 6:**
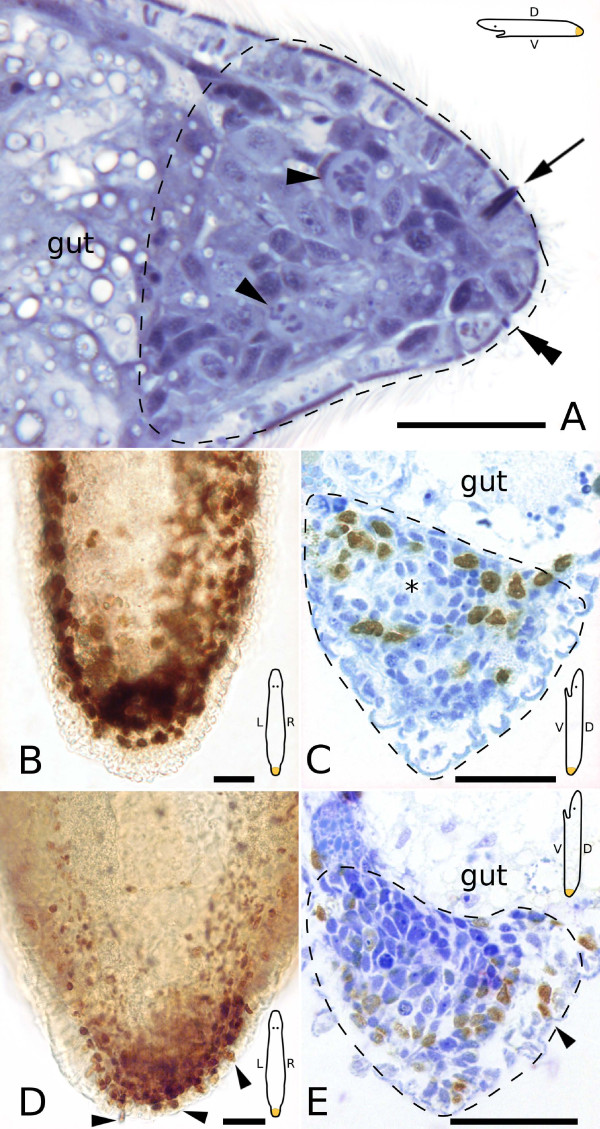
**Semithin sections and whole mounts of 48 hour tail plate blastemas**. Schemes are indicating the orientation of the animal in the respective subpanels. (A) At the anterior end (left), the blastema is delimited by the gut. Note the mitoses (arrowheads) within the blastema. An early duo-gland adhesive system, with a cell body close to the epidermis, has been regenerated (double arrowhead). Arrow points at a rhabdite gland. Stained after Heidenhain. (B, C) BrdU pulse. Accumulation of labeled cells in the blastema in whole mount (B) and section (C). Asterisk marks a label-free spot, the presumptive point of origin of the male genital apparatus. (B, C) from the same animal. (D, E) BrdU pulse-chase. (D) Labeled cells are concentrated in the blastema, and also appear in the epidermis (arrowhead). (E) Section of a different animal. Arrowheads in (D, E) point at labeled epidermal cells. Dotted lines indicate blastema extension. (C, E) stained with methylene blue. Scale bars are 20 μm.

The cell count of 48 hour blastemas in serial sections had more than doubled compared to a 24 hour blastema, amounting to 922 ± 79 (n = 5) cells, not counting newly built epidermal cells. The average dimension in the anterior-posterior axis was 46 ± 6 μm, in the dorso-ventral axis 59 ± 8 μm and in the left-right axis 46 ± 4 μm in sections (n = 5). All 48 hour blastemas were in a caudal, not in a ventral position (Figs. 4C, D, 6).

An average of 16 ± 4 (n = 17) mitoses was found in serial sections (Fig. 6A) and in fluorescent confocal stacks (see Additional file 1B). 49% of all mitoses were located at the anterior border and 41% in the central area of the blastema – few (10%) mitoses were found at the posterior tip. Only 16% of all mitoses were situated in the dorsal area of the blastema. In a BrdU pulse experiment (Fig. 6B, C), 236.6 S-phase cells were counted in serial sections, constituting 28% of all blastema cells. About one quarter (24%) of labeled cells were located in the dorsal blastema region. In BrdU pulse-chase experiments (Fig. 6D, E), the average number of S-phase cells found in serial sections was 363 ± 34 (n = 2), which is a fraction of 39% of all blastema cells.

BrdU pulse-chase experiments revealed several labeled cells in the epidermis after 48 hours (Fig. 6D, E). 22 ± 10 (n = 7) regenerated duo-gland adhesive systems were found in live animals and in serial sections (Figs. 4D, 6A), none of which showed BrdU label at this time point. Rhabdites were found interspersed between epidermal cells throughout and also at the caudal tip of the blastema, the former wound area (Fig. 6A). In one animal, the first presumptive signs of the regenerating male genital apparatus appeared. In the area of the emerging copulatory organ (i.e. the organ primordium) of a BrdU pulse animal, no labeled cells were present (Fig. 6C). No other formation of cells or clusters were apparent in serial sections of 48 hour regenerates.

#### 72 hours after amputation

**Figure 7:**
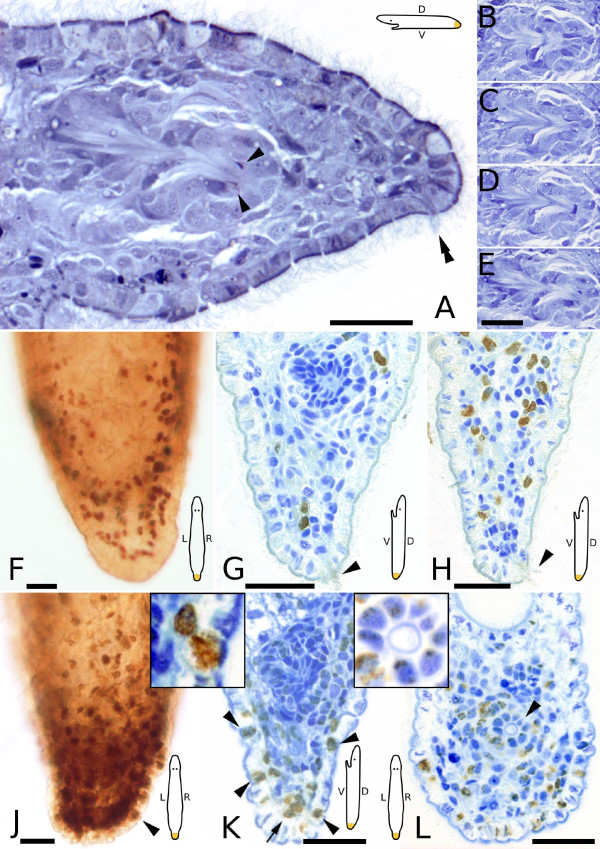
**Semithin sections and whole mounts of 72 hour tail plate blastemas**. Schemes are indicating the orientation of the animal in the respective subpanels. (A-E) Stained after Heidenhain, all pictures from the same series. (A) A regenerated duo-gland adhesive system *dg *is visible (double arrowhead). Arrowheads denote the regenerated tip of the male genital apparatus *mg*, consecutive sections showing the composition of this organ in (B-E). (F-H) BrdU pulse, all pictures from the same animal. (F) Whole mount. No accumulation of labeled cells. (G) Section in the area of the regenerating *mg *(densely blue stained area at the top). No labeled cells (brown) are present in the *mg*. (H) Section showing a number of labeled cells aside from the *mg*. (J-L) BrdU pulse-chase. (J) Whole mount, arrowhead points at a labeled epidermal cell. The accumulation of neoblasts in the blastema is evident. (K) Same animal as (J), note the labeled epidermal cells (arrowheads, inset) and the labeled nuclei of the *dg *(arrow). The regenerating *mg *is located at the top and shows freckled label in many cells (also see right inset). (L) Horizontal section of a different animal, arrowhead points at the tip of the regenerating *mg*. (G, H, K, L) stained with methylene blue. Scale bars are 20 μm, (B-E) same scale bar.

On average, a 72 hour blastema was made of 1422 ± 181 (n = 4) cells, excluding epidermal cells. Epidermal cells were counted in serial sections stained after Heidenhain, totalling 960 cells in a three day old blastema. In the anterior-posterior axis, the fixed blastema extended on average 84 ± 19 μm, in the dorso-ventral 61 ± 15 μm and in the left-right axis 56 ± 19 μm (n = 4) (Fig. 7A).

On average, 7 ± 2 mitoses were found per blastema (n = 6) in serial sections and confocal stacks. Most mitoses were found in the anterior (46%) or middle parts (48%) of the blastema. Regarding the dorso-ventral axis, most mitoses occured in the ventral (48%) or central (34%) rather than the dorsal area (18%). In a BrdU pulse experiment, labeled cells contributed to 10% (158 cells) to the blastema, no longer showing a dense accumulation of S-phase cells (Fig. 7F–H). As already seen in 48 hour blastemas, the growing anlage of the male genital primordium showed no label in BrdU pulse experiments (Fig. 7G). In BrdU pulse-chase experiments (n = 2), half (50% or 629.0 cells) of all blastema cells were labeled, including most cells of the male genital primordium (Fig. 7J–L). An increased number of epidermal cells was labeled in BrdU pulse-chase experiments compared to 24 and 48 hour blastemas. The label in cells of the genital primordium was generally weaker than in epidermal cells, often showing a sparse, freckled distribution.

After three days of regeneration, the differentiation of the largest organ system of the tail plate, the male genital apparatus, was at full activity. The tip of the stylet was already visible *in vivo *(Fig. 4E) as well as in sections (Fig. 7A, L). Attached to the base of the stylet was the developing vesicula granulorum lined with muscles (Fig. 7A–E). Long sensory cilia appeared at the caudal tip. A large number of duo-gland adhesive systems (51.0 ± 9.9, n = 3) was regenerated and could already be used for attaching to the substrate (see also Figs. 4F, 7A, G, H). Large gland cells with sizable secretion granules were already restored in the tail plate. The first duo-gland adhesive systems with labeled nuclei were found at this time point (Fig. 7K).

#### Further regeneration

Four to five days after amputation of the tail plate, the stylet grew to full size, and at the same time the first sperm appeared in the rebuilt seminal vesicle, connected via the ductus ejaculatorius with the vesicula granulorum. In this time, *M. lignano *was able to restore the basic functionality of the tail plate, that is the ability to copulate and to transfer sperm, and the ability to effectively attach to the substrate. The further regeneration of the duo-gland adhesive systems happened only gradually and took between 2 and 6 more days (see also [26,27]). Thus, the regeneration of the full number (about 130) duo-gland adhesive systems can be used as a final marker for complete regeneration.

## Discussion

### Cryo-based specimen preparation

For the first time, rapid cryo-immobilisation by means of high-pressure freezing was used as a primary fixation technique for ultrastructural preparations in flatworms. Excellent fine structure preservation was achieved, comparable to published data on *Caenorhabditis elegans *and other multicellular organisms (see [40,45] for reviews). Semi- and ultrathin sections from these preparations provided a good reference for evaluating the quality of chemically fixed samples of *M. lignano*, including our classification and counting of cell types. Generally, the morphological features reported from conventionally fixed samples (e.g. [43]) could be confirmed by this novel approach. In addition, the more reliable preservation of fine structure and antigenicity obtained with rapid freezing bears a great potential to allow more detailed insights into flatworm morphology. Finally, natively cryo-immobilised specimens should facilitate immuno-labeling of fixation-sensitive and/or easily translocated constituents [46].

### Wound healing and blastema formation

The wound is closed about 2 hours after amputation by flattening of surrounding epidermal cells (Fig. 3A), but is still susceptible to pressure and will break open easily during the first 4–8 hours. Compared to other taxa, e.g. holothurians, which require 2–3 weeks for wound healing [47], wound healing in flatworms is a very rapid process. The flattening of epidermis cells was already described for *M. lignano *[25] and is a common process also found in many other flatworms, among them catenulids [48], triclads [20], rhabdocoels [24], and other macrostomorphans [21]. A tail plate blastema is readily apparent 24 hours after amputation in *M. lignano*, but is most likely already present some time earlier. As the early blastema is located at the ventral side of the animal due to the contraction of ventral longitudinal muscle fibers [25], it can not easily be observed *in vivo *until about 24 hours after amputation.

A regeneration blastema may be defined as the accumulation of undifferentiated (or dedifferentiated) cells at the wound site as a response to amputation or injury, covered by old and new epithelial cells. Injury, both by artificial amputation and by fission for asexual reproduction, is a stimulus for forming a blastema, although the molecular nature of the stimulus is still unknown [20]. One trigger causing the formation of a blastema is thought to be contact of the epidermis with the mesenchyme or the gut, or the dorso-ventral interaction of the epidermis at the wound site (see literature in [20]). However, intercalary regeneration (if the middle piece is removed and the anterior and posterior end are brought together) is not relying on these triggers [49].

In *M. lignano*, the tail plate blastema (as an accumulation of proliferating stem cells) changes into differentiating organ primordia at about 72 hours after amputation, when the majority of amputated body parts have already been regenerated and the ratio of S-phase and mitotic neoblasts decreases compared to earlier blastemas.

### Indication of late determination or redetermination in neoblasts

In BrdU pulse-chase experiments with *M. lignano*, intact animals are incorporating BrdU into S-phase cells. Still, in serial sections it is evident that many of the cells labeled prior to amputation have become part of the blastema. This is especially obvious in the case of the male genital apparatus, whose cells are almost completely labeled in a pulse-3-days-chase experiment. An intact animal is not required to rebuild the genital apparatus, and after amputation of the tail plate only neoblasts distant from the genital apparatus remain. It appears unlikely that these distant S-phase cells were already committed to become part of the genital apparatus prior to entering S-phase.

Possible explanations are that these cells were not yet committed to follow a specific fate at replication time, or that they were committed, but were able to be redetermined during or after S-phase to contribute to the genital apparatus.

In an interesting experiment using *Dugesia lugubris*, a triclad with mixed ploidy (the same individual is characterized by different, but specific ploidies in testes, ovaries and somatic cells), it was shown that cells originally belonging to the gonads were contributing to building somatic tissue within the blastema [50], i.e. these gonadal cells were either dedifferentiated or, more likely, redetermined. Expression patterns of molecular markers led Agata and Watanabe [51] to suggest that neoblasts are already determined before going into S-phase in triclads, but lack of S-phase labeled cells makes interpretation of these results difficult.

### Neoblasts are not replicating at the place of organ differentiation

None of the cells in the developing male genital apparatus were labeled in BrdU pulse experiments, neither after two days (Fig. 6C), nor after three days (Fig. 7G) of regeneration. Not a single mitosis was observed in this organ system. In a pulse-72-hours-chase experiment, all cells of the male genital apparatus were labeled, suggesting that the progenitor cell(s) of this organ undergo replication – but not at the place of organ differentiation. Replicating or dividing cells were absent in the epidermis as well. Epidermal replacement occurs by migration of mesodermally located neoblasts into the epidermis in *Macrostomum *and in triclads [1,2]. The present study indicates that migration of determined neoblasts is not only essential in epidermal replacement, but also in the formation of complex organs such as the male genital apparatus.

### Progenitor cells of complex organs might undergo multiple divisions before differentiation

In sections of BrdU pulse-3-days-chase experiments, nuclei of differentiating epidermal cells and cells of the duo-gland adhesive system exhibit a stronger label than cells contributing to the male genital apparatus (Fig. 7K, L). Labeled epidermal progenitors seem to divide just once and to differentiate directly into an epidermal cell without further proliferation, recognizable by a strong BrdU label of the nucleus. Cells contributing to the male genital apparatus, however, are weakly stained compared to epidermal cells (Fig. 7K, L) and S-phase neoblasts (Fig. 7G, H). Provided the differently labeled nuclei are not a staining artefact, this is an indication that neoblasts contributing to the genital organ are going through several S-phases, thus dispersing the BrdU label among a couple of daughter cells. Another explanation for weakly stained nuclei of cells contributing to the genital apparatus is a slower S-phase of its progenitor cells, thereby taking up less BrdU than e.g. epidermal cell progenitors. However, we observed such a weak, spot-like label only in pulse-chase, and not in pulse experiments, suggesting it is neither a staining artefact, nor the result of a slow S-phase.

### Cell proliferation and differentiation in the blastema

After three days chasing time, cells labeled in S-phase before amputation and their progeny supply 50% of all blastema cells. The migration patterns of neoblasts into the blastema are not elucidated, but a previous study found that the response of the stem cell system to amputation in *M. lignano *is a local event restricted to the body regions near the wound [22].

In BrdU pulse-chase experiments, the first labeled epidermis cells covering the blastema appeared after two days, suggesting that about two days are required for replicating neoblasts to differentiate into an epidermal cell. However, newly built epidermal cells covering the blastema can already be observed after one day of regeneration (Fig. 5A). Similarly, duo-gland adhesive systems appeared in the blastema 48 hours after regeneration, whereas the first labeled duo-gland adhesive systems only appeared after 72 hours in pulse-chase experiments. The earliest differentiated cells in the blastema are likely the progeny of a pool of neoblasts arrested in G2-phase [29,30], which are activated for tissue maintenance and also for regeneration [22].

The fraction of blastemal cells in S-phase is reduced from almost 40% at 24 hours to about 30% at 48 hours, and drops to only 10% 72 hours post-amputation. This and the ongoing differentiation of the male genital apparatus from its primordium hint at the eventual breakup of the blastema as an accumulation of undifferentiated cells at this time point.

### Rate of regeneration

The caudal regeneration blastema in *M. lignano *is apparent 24 hours after amputation and grows rapidly by proliferation of neoblasts within the blastema. The regeneration process in this small flatworm can be readily quantified: during the first three days, 400–500 blastema cells are regenerated each day, starting with about 420 regenerated cells at 24 hours, 920 cells at 48 hours and 1420 cells at 72 hours after amputation. If replaced epidermal cells are included in the cell count, then 75% of the amputated tail plate, consisting of about 3,100 cells, have been replaced 72 hours after amputation. The largest organ of the tail plate, the male genital apparatus, is fully restored 4–5 days post-amputation.

This regeneration speed and the capacity of the obligatory sexually reproducing species *M. lignano *to regenerate reflects a likely adaptive response to known parasites and other environmental factors leading to the loss of body parts in their natural habitat [52]. Asexually reproducing flatworms often show a higher regenerate rate than *M. lignano*: for instance, the macrostomorphan *Microstomum lineare *and the catenulid *Stenostomum leucops *only need 1–2 days for regeneration of the head [21,53]. A particularly fast regenerating triclad is *Dugesia tahitiensis*, where 44% of all cells were described to be neoblasts or neoblast-like cells and which is reported to regenerate a head 2.5 days after regeneration [54].

### Mitotic activity within the blastema

The tail plate blastema in *M. lignano *shows distinct mitoses in anterior, middle and even posterior parts of the blastema at all observed time points. About half of these mitoses\are located at the anterior border of the blastema, near the stump, which seems to be a preferred site for cell divisions.

In a 24 hour blastema, the majority of S-phase neoblasts is located at the ventral side of the blastema, which is possibly connected with the location of the main nerve cords that are presumably responsible for directing the regeneration process [4,30]. The distribution pattern of neoblasts as found in the intact tail plate is reestablished after 3 or 4 days of regeneration.

Mitoses within the blastema have also been reported in an acoel flatworm [18]. In annelids [55] and nemerteans [7], mitotic divisions take place within the blastema as well. In crustaceans, the blastema grows through mitotic divisions of immigrated cells [9]. Deuterostomes that build a regeneration blastema show a similar spatial pattern regarding cell proliferation and mitoses: in crinoids, the blastema is one of the prefered sites of cell proliferation [56]. In urodeles, mitoses occur throughout the regeneration blastema, especially at the anterior border [11]. In teleost fish such as zebrafish, the blastema is formed by immigration of proliferating cells undergoing division within and at the base of the blastema [10]. Interestingly, blastemas in triclad flatworms have been described as lacking mitoses within the blastema itself, having mitoses only in the so-called postblastema, an area of the stump adjacent to the blastema [17].

## Conclusion

Flatworms are popular organisms for stem-cell and regeneration research due to a widespread and pronounced regeneration capacity and a likely totipotent stem-cell system even in adult animals. *Macrostomum lignano *is a well-suited model for quantitative regeneration studies, as it is comparably small and particularly amenable to cell proliferation markers and new fixation techniques such as high pressure freezing.

In this study we provide a detailled profile of cell proliferation and differentiation dynamics in the caudal blastema of the small flatworm *M. lignano*. We can clearly show that the blastema is a highly active center of proliferation, and that mitoses commonly occur within the blastema. This data set opens the way for successive studies on the migration of neoblasts into the blastema and the control of neoblasts in cell and tissue renewal. Comparative studies on blastema formation in other flatworms, particularly in triclads, are warranted to gain insights into common and derived patterns of regeneration in flatworms.

## Authors' contributions

BE amputated animals and labeled, fixed and embedded untreated and amputated animals, photographed sections, carried out cell counts, participated in high pressure freezing experiments and electron microscopy and drafted the manuscript. WS labeled, fixed and embedded animals and carried out semithin serial sections. MWH carried out high pressure freezing experiments and freeze substitutions, serial sections and electron microscopy. RG, KTN and RR helped conceive, design and discuss the experiments. ZA and MW helped discussing methods, results and drafting the manuscript. All authors, except RR, read and approved the final manuscript.

## Supplementary Material

Additional file 1 **Wholemount double-labeling of S-phase cells (pulse, green) and mitoses (red), superimposed on brightfield images**. Posterior part of the animal (A) 24 hours after amputation, (B) 48 hours after amputation. Note unspecific staining (red) of glands surrounding the female genital opening *fgo*. Scale bar is 50 μm for both panels. Click here for file

Additional file 2 **Morphology of epidermal cells labeled with BrdU after a 30 min pulse and 2–3 days chase**. (A) 2 days chase. Arrowhead points at a labeled round nucleus, contrasting to the typically lobulated nuclei of already differentiated epidermal cells (see also Fig. 2E). (B-H) 3 days chase. (C, E, G) bright field, (D, F, H) interference contrast images. (C-H) Arrowheads points at labeled nucleus of an epidermal cell with dark cytoplasm and comparatively short cilia. Scale bars are 5 μm, same scale bar for (C-H). Click here for file
