# Supplementary figures and images for: The caudal regeneration blastema is an accumulation of rapidly proliferating stem cells in the flatworm Macrostomum lignano
Source: BMC Dev Biol. 2009 Jul 15;9:41. doi: 10.1186/1471-213X-9-41 (PMC2717932; doi:10.1186/1471-213X-9-41)

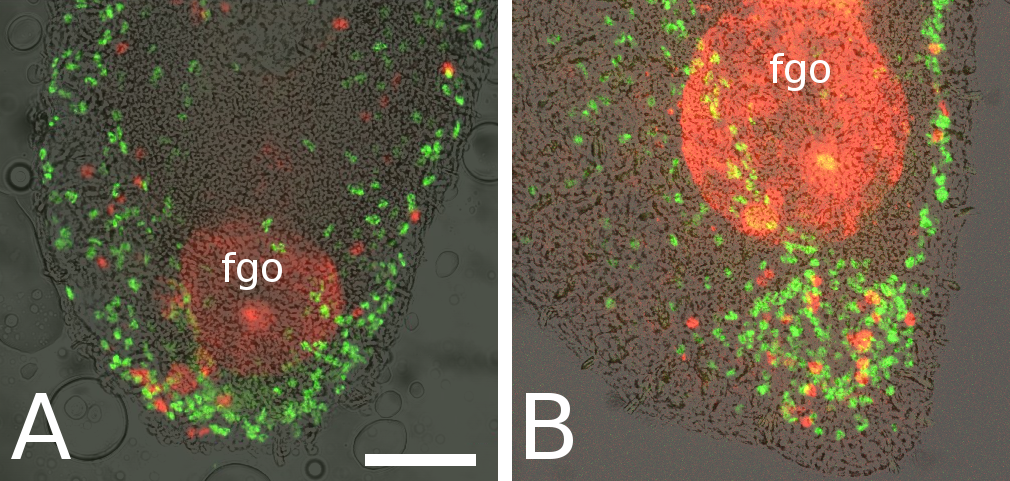

Supplement: Additional file 1 — Wholemount double-labeling of S-phase cells (pulse, green) and mitoses (red), superimposed on brightfield images. Posterior part of the animal (A) 24 hours after amputation, (B) 48 hours after amputation. Note unspecific staining (red) of glands surrounding the female genital opening fgo. Scale bar is 50 μm for both panels. [file 1471-213X-9-41-S1.tiff]

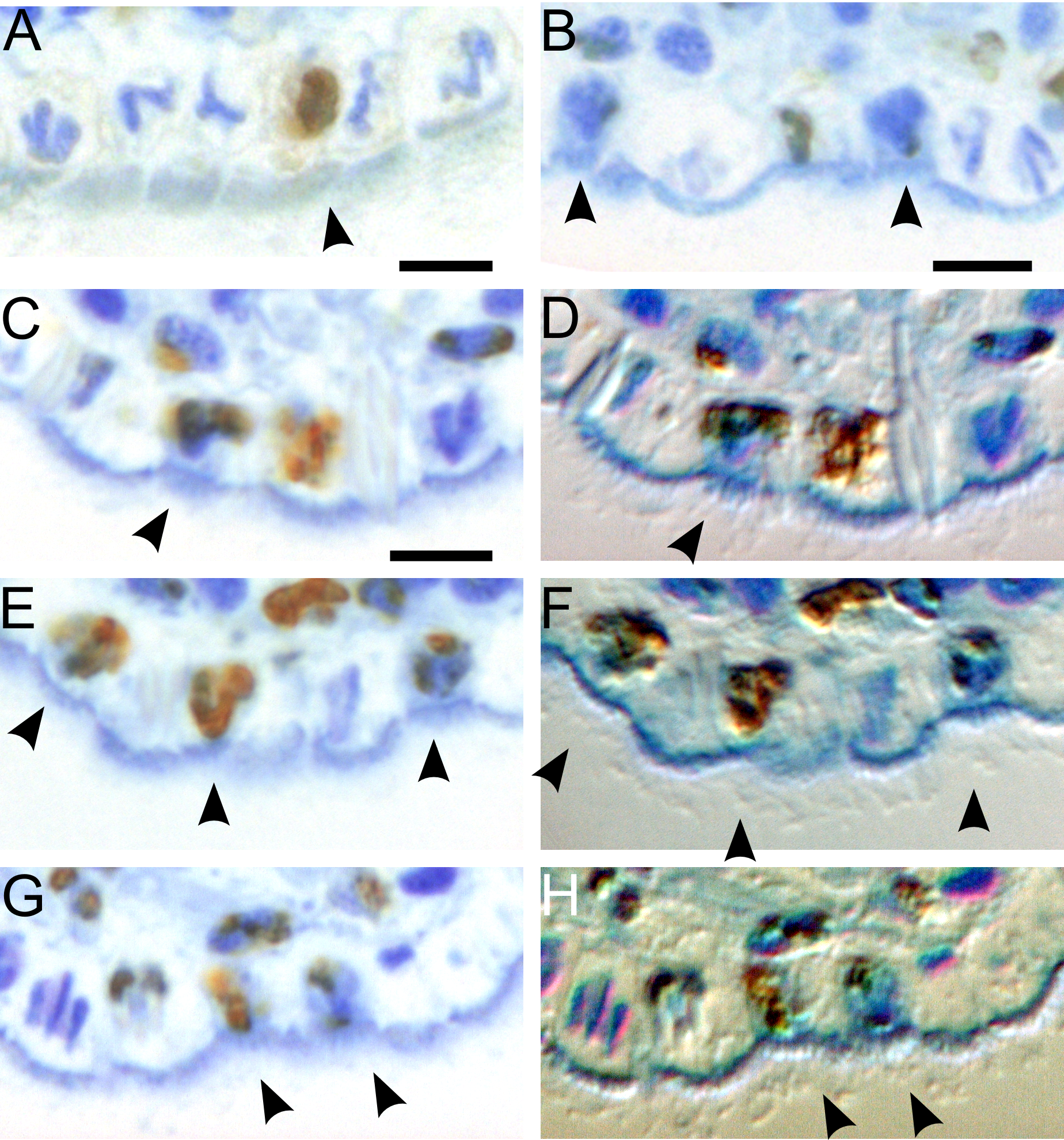

Supplement: Additional file 2 — Morphology of epidermal cells labeled with BrdU after a 30 min pulse and 2–3 days chase. (A) 2 days chase. Arrowhead points at a labeled round nucleus, contrasting to the typically lobulated nuclei of already differentiated epidermal cells (see also Fig. 2E). (B-H) 3 days chase. (C, E, G) bright field, (D, F, H) interference contrast images. (C-H) Arrowheads points at labeled nucleus of an epidermal cell with dark cytoplasm and comparatively short cilia. Scale bars are 5 μm, same scale bar for (C-H). [file 1471-213X-9-41-S2.tiff]
